# Influence of supplemental parenteral nutrition approach on nosocomial infection in pediatric intensive care unit of Emergency Department: a retrospective study

**DOI:** 10.1186/s12937-015-0094-0

**Published:** 2015-10-06

**Authors:** Dan Wang, Xiaoquan Lai, Chenxi Liu, Yuqi Xiong, Xinping Zhang

**Affiliations:** 1 School of Medical Management and Health Management, Tongji Medical College of Huazhong University of Science and Technology, Wuhan, China; 2 Tongji Hospital, Tongji Medical College of Huazhong University of Science and Technology, Wuhan, China

**Keywords:** Supplemental parenteral nutrition, Critically ill children, Nosocomial infection

## Abstract

**Background & aims:**

Nutritional support for patients in the intensive-care unit (ICU) is a part of standard care which promotes medical quality and decreases nosocomial infection. Supplemental parenteral nutrition (SPN) approach (enteral nutrition (EN) combined with parenteral nutrition (PN) when EN alone is insufficient) has become one major concern in nutrition research field. This research aims to explore the following relationships: (i) the relationship between SPN and nosocomial infection, (ii) the relationship between early and late SPN initiation and the development of nosocomial infection.

**Methods:**

A retrospective study was conducted in patients who met the inclusion criteria from February 2012 to February 2015 in Pediatric ICU (PICU). Patients were classified into two groups according to nutrition delivery approach-SPN group and EN alone group. Then SPN group were further divided into two subgroups by initiation timing, which were defined as early-initiation SPN and late-initiation SPN group respectively. Age, gender, serum albumin at admission, severity of disease, length of stay in PICU, nutrition delivery approach, amounts of delivered caloric intake and occurence of nosocomial infection were recorded. Univariate analysis and binary logistic regression analysis were performed to identify the risk factors and assess the independent effect of SPN approach on nosocomial infection in PICU of Emergency Department.

**Results:**

204 patients were included in our study. Compared with EN alone group, patients delivered by SPN approach had a higher nosocomial infection rate (34.0 vs.10.9 %, *p* < 0.001). The late-initiation subgroup of SPN approach was found to be an independent predictor of nosocomial infection in the logistic regression analysis model (OR = 3.40; 95 % CI, 1.13 ~ 10.19; *p* = 0.029). Serum albumin at admission (OR = 0.91; 95 % CI, 0.84 ~ 0.97; *p* = 0.008), mechanical ventilation (OR = 3.85; 95 % CI, 1.43 ~ 10.39; *p* = 0.008), severity of disease (OR = 3.79; 95 % CI, 1.03 ~ 13.99; *p* = 0.045) and PICU length of stay (OR = 1.23; 95 % CI, 1.11 ~ 1.35; *p* < 0.001) were also identified as significant risk factors for nosocomial infection.

**Conclusions:**

Our study shows late-initiation SPN approach increases the incidence of nosocomial infection compared with early-initiation approach in critically ill children in PICU of Emergency Department. Compared with EN alone group, patients delivered by SPN approach had a higher nosocomial infection rate.

## Background

Nutritional support is vital for patients in the intensive-care unit (ICU). Despite increasing awareness of nutrition support, malnutrition is still prevalent in Pediatric ICU patients, with reports in the range of 25 to 45 % at the time of admission [1–3]. Malnutrition has been identified as an independent factor for higher rate of nosocomial infections, which has become one of the most severe clinical outcomes associated with substantial morbidity and mortality, prolonged hospital stay and increased economic burden [4, 5]. Therefore, it is of great significance to explore nutrition support for pediatric patients with nosocomial infection being the main focus of clinical outcome.

Driven by improving clinical outcomes and decreasing complex complications, nutrition delivery approach has been a major concern in nutrition research field for decades. Early enteral feeding approach has been preferred based on several guidelines [6–8]. In addition, substantial evidence showed enteral nutrition (EN) approach has been associated with improved clinical outcomes compared with parenteral nutrition (PN) approach [9, 10]. However, critically ill patients often can not meet their energy target due to various situations and interruptions, such as medical investigations, surgery or gastrointestinal intolerance [11]. Therefore, PN approach was taken for critically ill patients. Although energy balances can be achieved by PN approach in short terms, most published studies showed PN approach resulted in increased infectious complications [12, 13]. Under this setting, researchers started to explore SPN approach (EN combined with PN when EN alone is insufficient) as one combination approach. But what are the benefits and potential harm of SPN approach compared to EN approach is still unknown when usual nutrition target is not met. And previous studies have put forth conflicting recommendations about SPN approach. The findings of Heidegger, Villet and Claude Pichard support potential value of SPN as one effective approach to reduce nosocomial infections [14–16], whereas some other studies suggest opposite conclusion and recommend that PN not be used combined with EN [17, 18].

Moreover, as timing of nutrition support being an important factor which would contribute to clinical outcomes, it was absent or poorly controlled in previous studies. Guidelines recommend early initiation of EN approach within 48 h at admission [8, 9, 12]. However, optimal timing for the initiation of PN still remains controversial [19]. Whether early or late-initiation of SPN approach influence the incidence of nosocomial infection is worth further exploring.

In this study, we aimed to explore two relationships under the current practice in PICU of Emergency Department: (i) the relationship between SPN and nosocomial infection, (ii) the relationship between early and late SPN initiation and the development of nosocomial infection.

## Methods

### Study patients

This retrospective study was conducted in PICU of Emergency Department which enrolled non-surgical patients in a tertiary hospital in Wuhan, China. The tertiary hospital covered over 4000 beds and reached 4280,000 outpatients and inpatients amounts in 2014. All patients aged younger than 18 years were enrolled on admission to the hospital PICU of Emergency Department for longer than 48 h and could tolerate some amount of enteral nutrition from February 2012 to February 2015. The patients who had more than one PICU admission during hospitalization, or had been referred to the PICU from other wards in the hospital and already received EN or PN, or were considered ineligible for EN by the physician due to contraindications of EN support during the PICU stay (including complete intestinal obstruction, necrotizing enterocolitis, gastrointestinal dysfunction caused by failure, severe infection, trauma and postoperative digestive tract paralysis and high-flow intestinal fistula) were excluded for this study. The study was approved by Ethics Committee of Tongji Medical College, Huazhong University of Science and Technology (IORG: IORG0003571).

### Data collection

Patients’ demographic and clinical characteristics were obtained from the Hospital Information System (HIS). These included sex, age, admittance date, serum albumin at admission, severity of disease, mechanical ventilation, invasive diagnosis operation and PICU length of stay. The nutritional data for the date that nutritional support was initiated, daily caloric intake and occurrence of nosocomial infection was also collected.

### Definition of nutritional support groups

Two groups of patients were generated based on their nutrition delivery approach. The EN alone group included patients who only received EN while the other group received SPN support combined with EN during the PICU stay. To control the influence of timing, patients in SPN approach were further classified into two subgroups. Subgroup 1 included patients who initiated SPN in 48 h at admission, which was defined as early-initiation group. Subgroup 2 defined as late-initiation group included patients who initiated SPN between 2th day and 6th day of PICU stay. The physicians determined nutritional support for patients in PICU of Emergency department for most cases guided by standard operation procedure [20], and consultation of the pediatric nutritionist was initiated in complex cases only.

If patients had functioning gastro-intestinal tract and were anticipating to remain unable to take oral nutrition for more than 3–7 days, EN would be given. Enteral calories were delivered by oral diet, nasogastric or nasojejunal tubes, and the feeding tubes were inserted by trained nurses. EN was delivered by feeding drip at a constant speed of 10–20 ml/kg/d. The volume of gastric residual was measured 4 to 6 times a day. If the clinician felt that the children could not achieve the target caloric intake (100 % caloric requirement per day) by enteral feedings alone for more than 5 days, SPN would be delivered in our study. Parenteral calories were delivered in a mixed way by ready-to-mix 3-chamber bag (Kabiven G11 %, Fresenius Kabi AB Sweden) or by the physicians’ preparation containing amino acids, glucose, lipids, and electrolytes, and trace elements, minerals, and vitamins were added as clinically appropriate. We characterized patients as “enteral tolerant” if they received ≥1000 enteral calories/kg/day at any point within the PICU stay. The threshold for “enteal tolerant” was one arbitrary selection for the “enteral tolerant” intake requirements used by the attending physicians in the hospital. Predicted energy expenditure (PEE) calculated by the WHO equation [21] and daily caloric intake abstracted from the HIS was used for our analyses.

### Definition for some risk factors

Age groups were classified by the criteria of American Centers for Disease Control and Prevention [22]. Severity of disease was scored into two levels by the judgment of physicians with a comprehensive evaluation of patient’s condition, nursing and treatment plans and clinical emergency, such as coma or shock [23]. Serum albumin at admission was taken as one indicator for primary nutrition evaluation. Invasive diagnosis operation was defined as invasive examinations which concluded lumbar puncture, sternal puncture and abdominal puncture [24].

### Study outcomes

The primary study outcome was the incidence of nosocomial infection. Infections were defined according to the definition from Chinese Diagnostic Criteria for Nosocomial Infection [25]. Five infection categories were defined: pneumonia(ventilator or non-ventilator-associated pneumonia, and other lower respiratory tract infections), bloodstream infection(laboratory-confirmed bloodstream infections and clinical sepsis); urogenital infection(device-associated or non-device associated urinary tract and genital infection); abdominal infection(intra-abdominal infections); and other infection [14].

### Statistical analysis

Continuous variables such as age and weight were compared using a Students’ *T*-test or Mann–Whitney U; chi-square and Fisher’s exact test were used to compare categorical variables such as timing of PN initiation and sex. Univariate analysis was used to compare these factors for patients with and without nosocomial infection. Binary logistic regression was used to evaluate the independent impact of nutrition support approach and identify the independent impact of risk factors on nosocomial infection. Study variables which were significant at a 2-tailed *p* < 0.05 in the univariate analysis or considered important were entered into the binary logistic model. Normality and homoscedasticity were checked previous to the implementation of parametric statistical tests. Variables which did not follow normal distribution were conducted for logarithmic transformation. Statistical analyses were performed with SPSS 12.0 (SPSS Inc., Chicago, IL, USA).

## Results

**Fig. 1:**
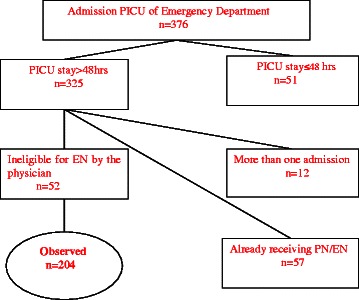
Flow diagram of patient recruitment. A total of 376 pediatric patients aged younger than 18 years were enrolled on admission to the hospital PICU of Emergency Department. Of the 376 pediatric patients, 51 patients were discharged within 48 h, 12 patients were had more than one PICU admissions during hospitalization, and 57 patients were referred from other wards in the hospital and already received enteral nutrition (EN) or parenteral nutrition(PN). These patients were excluded from the study for exclusion criteria. Besides, 52 patients judged by the physicians as ineligible for EN due to the contraindications were also excluded from this study, and 204 patients were enrolled in the study finally

During the 3-year study period 204 children were enrolled in our research. The flow diagram of patient recruitment was presented in Fig. 1. Characteristics of our study patients were listed in Table 1. There were 65.7 % male and 34.3 % female, and patient received 287.7 (179.3–411.3) kcal/ day. With the 46 nosocomial infection cases, pneumonia and bloodstream infection took the majority (56.52 and 34.78 % for pneumonia and bloodstream infection respectively).

**Table 1:** Characteristics of study patients (*n* = 204)

Characteristics	*n* (%)/median (IQR)
Gender, *n* (%)	
Male	134 (65.7)
Female	70 (34.3)
Age (month), median (IQR)	9.0 (3.0–30.0)
Weight (kg), median (IQR)	8.1 (5.0–13.0)
Severity of disease, *n* (%)	
Class 1	86 (42.2)
Class 2	118 (57.8)
Caloric intake (kcal/day), median (IQR)	287.7 (179.3–411.3)
Nosocomial infection, *n* (%)	46 (22.6)
Type of nosocomial infection, *n* (%)	
Pneumonia	26 (56.5)
Bloodstream infection	16 (34.8)
Urogenital infection	3 (6.5)
Abodominal infection	1 (2.2)
Type of nutritional support, *n* (%)	
EN alone group	101 (49.5)
Early-initiation group	60 (29.4)
Late-initiation group	43 (21.2)

Note: *IQR* interquartile ranges (25th to 75th percentile)

Of the whole 204 study patients, 101 (49.5 %) received EN alone and 103 (50.5 %) received SPN. Their demographics, clinical characteristics, data for nutrition support and clinical outcome were presented in Table 2. Patients receiving SPN showed a significantly higher percentage of nosocomial infection than patients receiving EN alone (34.0 vs.10.9 %; *p* < 0.001). All patients in EN alone group were initiated within 48 h, while 58.3 and 41.7 % patients in SPN group were initiated within 48 h and between 2th day and 6th day respectively. For demographic factors, weight showed significant difference that children in SPN group were heavier than children in EN alone group. All clinical factors included showed significant difference in two groups. Patients in SPN group had lower level of serum albumin at admission, higher percentage of more severe condition (Class 2), mechanical ventilation, invasive diagnosis operation and longer PICU length of stay than those in EN alone group.

**Table 2:** Distribution of clinical outcomes, nutrition factors and other characteristics of patients

Characteristics	EN alone group (*n* = 101)	SPN group (*n* = 103)	Statistic value	*P* value	SPN subgroup1 (*n* = 60)	SPN subgroup2 (*n* = 43)	Statistic value	*P* value
Nosocomial infection, *n* (%)	11 (10.9)	35 (34.0)	15.57^a^	<0.001	15 (25.0)	20 (46.5)	5.17^a^	0.02
Total caloric intake (%), median (IQR)	64.1 (46.9–83.6)	64.8 (51.7–85.7)	−0.29*^b^	0.77	65.42 (50.8–82.9)	64.80 (52.4–86.1)	0.57^b^	0.57
Caloric intake (kcal/day), median (IQR)	284.1 (178.9–401.0)	299.5 (179.6–480.1)	5163.00^c^	0.93	313.6 (179.0–420.7)	268.9 (179.6–542.4)	0.19^b^	0.74
Gender, *n* (%)			0.04^a^	0.85			6.26^a^	0.01
Male	67 (66.3)	67 (65.0)			45 (75.0)	22 (51.0)		
Female	34 (33.7)	36 (35.0)			15 (25.0)	21 (49.0)		
Age (month), median (IQR)	9.0 (3.0–26.0)	9.0 (3.0–37.0)	3.44*^b^	0.07	11.0 (3.0–49.0)	7.0 (4.0–30.0)	0.17^b^*	0.86
Age groups, *n* (%)			2.34^a^	0.50			5.54^d^	0.07
Infants	58 (57.4)	55 (53.4)			31 (51.7)	24 (55.8)		
Toddlers	23 (22.8)	21 (20.4)			11 (18.3)	10 (23.3)		
Preschoolers	9 (8.9)	8 (7.8)			7 (11.7)	1 (2.3)		
Childhood and young teens	11 (10.9)	19 (18.4)			11 (18.3)	8 (18.6)		
Weight (kg), median (IQR)	8.2 (5.5–12.3)	8 (4.5–15.0)	5.00*^b^	0.03	8.8 (4.6–16.0)	8.0 (4.5–15.0)	0.12^b^*	0.91
Serum albumin at admission (g/L), mean ± SD	38.50 ± 6.30	35.94 ± 6.11	2.94^b^	0.004	35.95 ± 6.33	35.94 ± 5.86	0.01^b^	0.99
Severity of disease, *n* (%)			21.69^a^	<0.001			3.77^a^	0.05
Class 1	59 (58.4)	27 (26.2)			20 (33.3)	7 (16.3)		
Class 2	42 (41.6)	76 (73.8)			40 (66.7)	36 (83.7)		
Mechanical ventilation, *n* (%)	11 (10.9)	29 (28.2)	9.64^a^	0.002	15 (25.0)	14 (32.6)	0.71^a^	0.40
Invasive diagnosis operation, *n* (%)	18 (17.8)	35 (34.0)	6.92^a^	0.009	20 (33.3)	15 (34.9)	0.03^a^	0.87
PICU length of stay (day), median (IQR)	4.0 (3.0–7.0)	8.0 (5.0–14.0)	2922.00^c^	<0.001	7.5 (4.0–14.0)	9.0 (6.0–14.0)	−0.53^b^	0.59

Note: Variables with * were conducted for logarithmic transformation. Statistic values with ^a, b, c, d^ were Pearson’s chi-square value, Students’ t value and Mann–Whitney U value and Fisher exact test value respectively

Patient characteristics of two subgroups of SPN approach were also presented in Table 2. Subgroup 2 had a higher percentage of nosocomial infection than subgroup 1 (46.5 vs.25.0 %, *p* = 0.02). Gender showed significant difference with more males in subgroup 1. Age, age groups, weight and clinical factors showed no significant difference in two subgroups.

Univariate relationship between independent predictors and occurrence of nosocomial infection were reported in Table 3. The incidence of nosocomial infection was significantly different in relation to nutrition delivery approach: EN alone approach 23.90 %, early-initiation approach 28.30 % and late-initiation approach 47.80 % (*p* < 0.05). Compared with patients with non-nosocomial infection, patients with nosocomial infection had significantly lower level of serum albumin at admission, more severe conditions(Class 2), higher rate of mechanical ventilation and invasive diagnosis operation and longer PICU length of stay.

**Table 3:** Independent predictors for nosocomial infection

Characteristics		Univariate				Binary logistic regression		
		Non-nosocomial infection (*n* = 158)	Nosocomial infection (*n* = 46)	Statistic value	*P* value	OR	95 % CI	*P* value
Nutrition route, *n* (%)				22.80^a^	<0.05			
EN only group		90 (57.0)	11 (23.9)					0.090
SPN group:	Subgroup1	42 (26.5)	13 (28.3)			3.40	1.13 ~ 10.19	0.029
	Subgroup2	26 (16.5)	22 (47.8)			1.87	0.56 ~ 6.22	0.281
Total caloric intake (%), median (IQR)		65.0 (48.6–84.0)	63.5 (50.9–82.8)	0.02*^b^	0.98			
Caloric intake (kcal/day), median (IQR)		285.3 (170.5–400.6)	302.8 (222.2–509.3)	1.77*^b^	0.08			
Gender, *n* (%)				1.78^a^	0.18			
Male		100 (63.3)	34 (73.9)					
Female		58 (36.7)	12 (26.1)					
Age (month), median (IQR)		8.5 (3.0–30.0)	10.0 (4.0–54.0)	1.39*^b^	0.17			
Age groups, *n* (%)				2.45^d^	0.48			
Infants		89 (56.30)	24 (52.20)					0.495
Toddlers		35 (22.20)	9 (19.60)			0.87	0.27 ~ 2.76	0.809
Preschoolers		14 (8.90)	3 (6.50)			0.61	0.09 ~ 4.19	0.602
Childhood and young teens		20 (12.70)	10 (21.70)			2.33	0.64 ~ 8.47	0.198
Weight (kg), median (IQR)		8.0 (5.0–12.6)	9.0 (6.0–17.5)	1.61*^b^	0.11			
Serum albumin at admission (g/L), median ± SD		38.19 ± 6.05	33.85 ± 6.16	4.26^b^	<0.001	0.91	0.84 ~ 0.97	0.008
Severity of disease, *n* (%)				27.27^a^	<0.05	3.79	1.03 ~ 13.99	0.045
Class 1		82 (51.90)	4 (8.70)					
Class 2		76 (48.10)	42 (91.30)					
Mechanical ventilation, *n* (%)		20 (12.70)	20 (43.50)	21.47^a^	<0.05	3.85	1.43 ~ 10.39	0.008
Invasive diagnosis operation, *n* (%)		30 (19.00)	23 (50.00)	17.82^a^	<0.05	1.49	0.55 ~ 4.07	0.436
PICU length of stay (day), median (IQR)		5.0 (3.0–8.0)	12.5 (8.0–15.3)	1172.5^c^	<0.001	1.23	1.11 ~ 1.35	<0.001

Note: Variables with * were conducted for logarithmic transformation. Statistic values with ^a, b, c, d^ were Pearson’s chi-square value, Students’ t value and Mann–Whitney U value and Fisher exact test respectively. *OR* odds ratio, *CI* confidence interval

To evaluate the independent effect of nutrition delivery approach on nosocomial infection, we conducted a binary logistic regression model. Variables included in initial regression were from study variable that were <0.05 in the univariate analysis or considered important. The results indicated that late-initiation SPN approach was associated with increased nosocomial infection. Serum albumin at admission, mechanical ventilation, severity of disease and PICU length of stay were also found as significant predictors of nosocomial infection (Table 3).

## Discussion

In this study, 46 nosocomial infection cases were included. With only four cases of other types of nosocomial infection, the majority of nosocomial infecteions were distrubuted in pneumonia and bloodstream infection. It was likely due to the condition that PICU of Emergency Department in the hospital excluded the surgical patients. We also found that late-initiation SPN approach was associated with an increased risk of nosocomial infection compared with early-initiation SPN approach, while the association between early-initiation SPN approach and nosocomial infection was not demonstrated.

Our results were similar to one observational study conducted by Matthew J Sena in critically ill trauma patients. Their findings validated the adverse effect of SPN approach in critically ill patients [17]. It showed an increased association between early SPN and nosocomial infection. But early SPN was defined as SPN initiated within 1 week, which was slightly different from our study, in which 48 h was set as criteria. Additionally, our findings do support other studies as for other risk factors from the binary regression analysis. Serum albumin at admission, mechanical ventilation and severity of disease showed significant association with nosocomial infection, and these risk factors have been demonstrated by other studies [26–29]. PICU length of stay was also found as one predictor on nosocomial infection [30]. Although our results were similar to the previous studies, we still need to be cautious about interpreting our results, because previous studies did not take into account of timing factor or took different definition for the timing factor.

However, our findings violate results of several previous studies and guidelines which recommend SPN as one effective way to reduce nosocomial infections [14–16, 31]. The possible factors for such disagreement are discussed as follows. Firstly, discrepancy in sample population may contribute to such difference. Sample population in our study was medical patients aged younger than 18 years while adult patients were chosen as study population in aforementioned studies. Children patients, as one group with specific physiological characteristics, were likely more sensitive to outside environment, such as intravenous -delivered route by SPN approach. Although studies which came to the same findings were also conducted in adult patients, children patients can not be ignored as one possible predictor for nosocomial infections. Based on such consideration, the variable of age groups which classified pediatric patients into four groups were analysed. However, the age groups showed no significant difference in both univariate and regression analyses. Considering the sample size and exclusion of adult patients in our study, future comparative studies can be done for exploring the difference between study patients. Another factor for our observation includes difference in caloric quantity. Previous studies showed different caloric quantities delivered by EN or SPN approach, in which one multi-center random controlled research designed combined feeding strategy with exact 100 % provision of energy target. It is still not certain exactly how an increased number of calories might influence infection rate, regardless of the composition of nutrition support.

Based on foregoing discussion, one most possible explanation for association between late-initiation SPN approach and nosocomial infection in our study is PN approach itself. The common finding is PN route is associated with an increase in postoperative complications. Such finding has been demonstrated by other studies previously [32, 33]. Moreover, SPN group and EN alone group received similar proportion of their target requirements in our study in which controlled the influence of nutrition amount (*p* > 0.05), which controlled the effect of nutrition amount on nosocomial infection. Therefore, increased risk of nosocomial infection by SPN approach is likely, at least in part, due to PN approach itself. Although some available guidelines recommend PN when caloric goals are not met during the first week, it has not been shown that this approach is beneficial. A multicenter observational study conducted in 2011 showed the most common reason for initiation of PN was “No reason” [34]. The anxiety of providing no nutrition support possibly impacted the choice of the delivery of PN which leaded most clinicians to select SPN, and it still plays an important role under current nutrition practice. One more possible explanation is timing of SPN initiation. Timing is one potential factor which is still under debate for PN initiation. Patients in early-initiation and late-initiation group showed different statistical results in binary logistic regression. Late-initiation SPN approach was likely associated with delayed energy balance, and negative energy balance has been validated to correlate with increased infections [15]. Under the circumstances of Emergency Department in our study, the need for quicker restoration of energy balance and important indices was likely more urgent, which may increase the risk for nosocomial infection.

There were several limitations in this study. Firstly, it was a retrospective study with a relatively small group of both the study population (*n* = 204) and the number of events (*n* = 46) in a single center, and this study is limited by the possibility that patients from SPN group and EN alone group differed systematically and the small size of events may preclude the observed difference to be significantly different. Thus the conclusion should be interpreted and applied with caution. Additionally, the study population only covered Chinese pediatric patients, which may limit the generalizability to other ethnic groups. Future studies should include a larger sample size and multiple institutions containing other ethnic groups. Secondly, our study was also limited due to some poorly defined concepts. Such concepts such as “adequate” or “sufficient” nutrition support and the criteria for “enteral tolerant”, “early” or “late” initiation remained unclear in existing literatures and guidelines. It is hard to reach the consensus about whether 48 h or 1 week maybe better set as criteria for “early” initiation. We define “early” initiation of SPN approach as within 48 h at admission, referring to early initiation of EN approach and the nutrition practice in our study institution, and the threshold for “enteral tolerant” was an arbitrary selection which was defined as receiving ≥1000 enteral calories/kg/day at any point during PICU stay. Our study was also limited due to a lack of data on nutritional assessment measures, such as Nutritional Risk Index (NRI) score, and Body Mass Index (BMI) was also not available for the missing data of height for majority of pediatric patients. Thus we took serum albumin at admission as the alternate indicator for primary nutrition evaluation. And further random controlled studies need to be designed for controlling potential confounders, such as nutritional practices.

Even though our study was limited in some aspects as one retrospective study, our findings provided the new insights with regards to controversial nutrition topic concerning SPN approach and its delivery timing. Further researches would be encouraged to further explore and validate the SPN approach and its other aspects.

## Conclusion

This study shows late-initiation SPN approach increases the risk of nosocomial infection compared with EN alone approach in PICU of Emergency Department. Serum albumin at admission, mechanical ventilation, severity of disease and PICU length of stay are important risk factors for nosocomial infections.

## Abbreviations

PICU: Pediatric intensive care unit
EN: Enteral nutrition
PN: Parenteral nutrition
SPN: Supplemental parenteral nutrition
HIS: Hospital information system
NRI: Nutritional risk index
BMI: Body mass index
